# A Unique Presentation of Ectopic Thyroid Tissue: Case Report and Management Principles

**DOI:** 10.7759/cureus.28717

**Published:** 2022-09-03

**Authors:** Vivek Sanker, Azeem Mohamed, Maanasi Pranala, Varghese Tharakan

**Affiliations:** 1 Surgery, Noorul Islam Institute of Medical Science (NIMS) Medicity, Trivandrum, IND; 2 General Surgery, Noorul Islam Institute of Medical Science (NIMS) Medicity, Trivandrum, IND; 3 Surgery, Believers Church Medical College Hospital, Pathanamthitta, IND; 4 General Surgery, Believers Church Medical College Hospital, Pathanamthitta, IND

**Keywords:** biopsy, hypothyroidism, swelling, submandibular sialadenitis, ectopic thyroid tissue

## Abstract

Ectopic thyroid is a rare clinical presentation to encounter in day-to-day clinical practice. It occurs due to developmental defects in the early stages of the thyroid gland embryogenesis during its descent from the floor of the primitive foregut to its final pre-tracheal position. It is usually present along the extent of the thyroglossal duct as well as in distant locations such as sub-diaphragmatic or mediastinal spaces. The diverse clinical presentation of this rare entity often causes a diagnostic dilemma. A thyroid scintigraphy scan is pivotal in the diagnosis of ectopy, but ultrasonography is done more frequently. Surgical management is preferred for symptomatic cases, followed by radioactive iodine ablation and levothyroxine supportive therapy for refractory cases. We present a case of a 62-year-old female patient who presented with pain and swelling of the right submandibular region. On ultrasonography, a 5*4 cm firm mobile swelling of the right submandibular region was found, suggestive of right submandibular sialadenitis. Fine needle aspiration cytology (FNAC) was subsequently done, and it showed features of basaloid neoplasm like pleomorphic adenoma, and as the thyroid tissue was in an ectopic location, it must have been misdiagnosed. The patient was then taken up for right submandibular sialoadenectomy, and the histopathological examination of the operative specimen showed nodular colloidal goiter and mild chronic sialadenitis. Ectopic thyroid can present at various anatomical locations and thereby has varied clinical presentations which makes it a diagnostic dilemma for clinicians. The usual radiological investigations done include USG and CT scan, whereas thyroid scintigraphy is more precise in reaching the diagnosis of ectopic thyroid. The confirmatory diagnostic method is the histopathological examination of the excised specimen. Most cases of ectopic thyroid are asymptomatic and require regular follow-up. Symptomatic cases are managed by surgical excision followed by periodic monitoring and adequate thyroxine replacement.

## Introduction

Ectopic thyroid tissue (ETT) is a rare entity with a prevalence of one in 1,00,000 - 3,00,000 among the general population and with an increased prevalence among patients with an underlying thyroid disorder [1,2]. Most cases occur due to a developmental defect during the period of embryogenesis of the gland. The usual location of ETT is in the midline, along the extent of the thyroglossal duct and the base of the tongue [3,4]. Unlike in this case, this entity can still occur even in various other distant locations in the body, such as in the subdiaphragmatic space, mediastinal space, pelvis, and also the thoracic cavity [5]. Thus it often becomes a diagnostic dilemma for clinicians because of its diverse clinical presentations. Patients suspected to have an ETT should have a proper pre-operative work-up, including ultrasonography, a computed tomography scan, and fine-needle aspiration cytology (FNAC) of the tissue [6]. It helps to ascertain the histological components of the tissue sample and also distinguish between a benign and a malignant mass. A thyroid scintigraphy scan, which aids in the diagnosis of ETT, is crucial in many cases. Symptomatic cases are treated surgically, which is curative as well. This case report turned out to be a diagnostic surprise as our patient initially presented as a case of submandibular sialadenitis.

## Case presentation

A 62-year-old female with a history of dyslipidemia and chronic obstructive lung disease on Seroflo metered dose inhaler (1mg, Gurugram, India) and HbsAg positive presented with pain and swelling of the right submandibular region, with a recent increase in size (Table 1). She also has a history of lumbar disc prolapse with radiculopathy in 2015. She denies any drug allergies, smoking, or alcohol consumption. 

**Table 1:** Initial laboratory test results

Laboratory test	Result
Haemoglobin (Hb)	14 g/dL
Erythrocyte sedimentation rate (ESR)	20 mm/hr
Random blood sugar (RBS)	144 mg/dL
HbA1c	6.20%
Thyroid-stimulating hormone (TSH)	1.44 uIU/mL
Triiodothyronine (T3)	1.94 pmol/L
Tthyroxine (T4)	12.3 pmol/L
Hepatitis B surface antigen (HbsAg)	3810.65 (Reactive)

**Figure 1:**
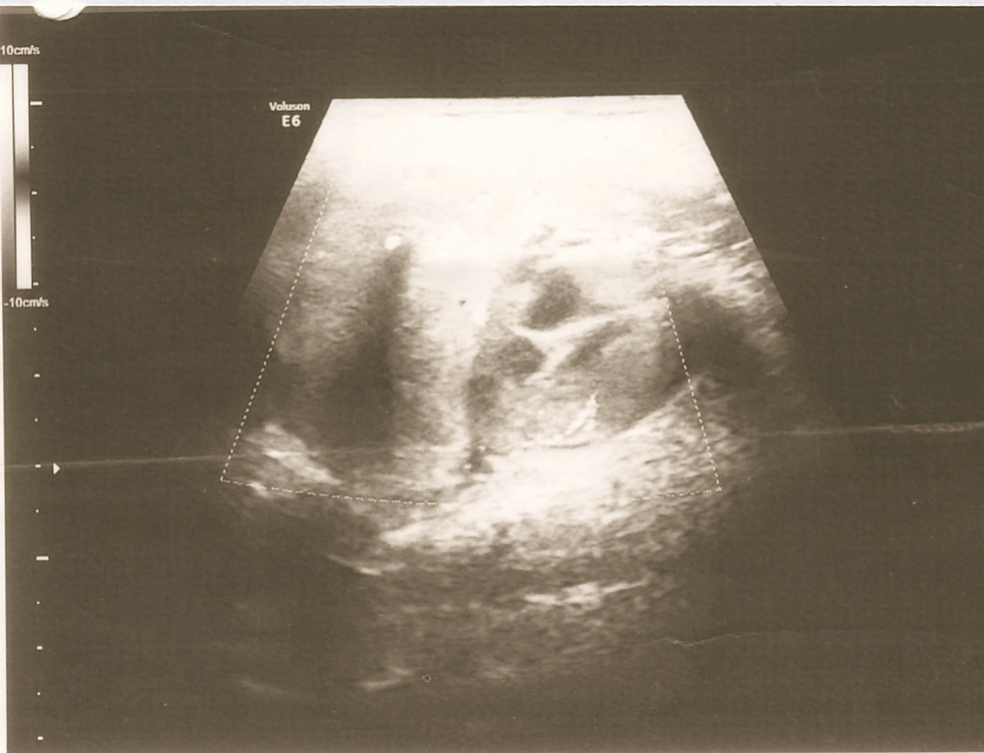
USG of the right submandibular region Heterogeneously rounded lesion measuring 5*3.5*4.2 cms with internal cystic areas and vascularity.

**Figure 2:**
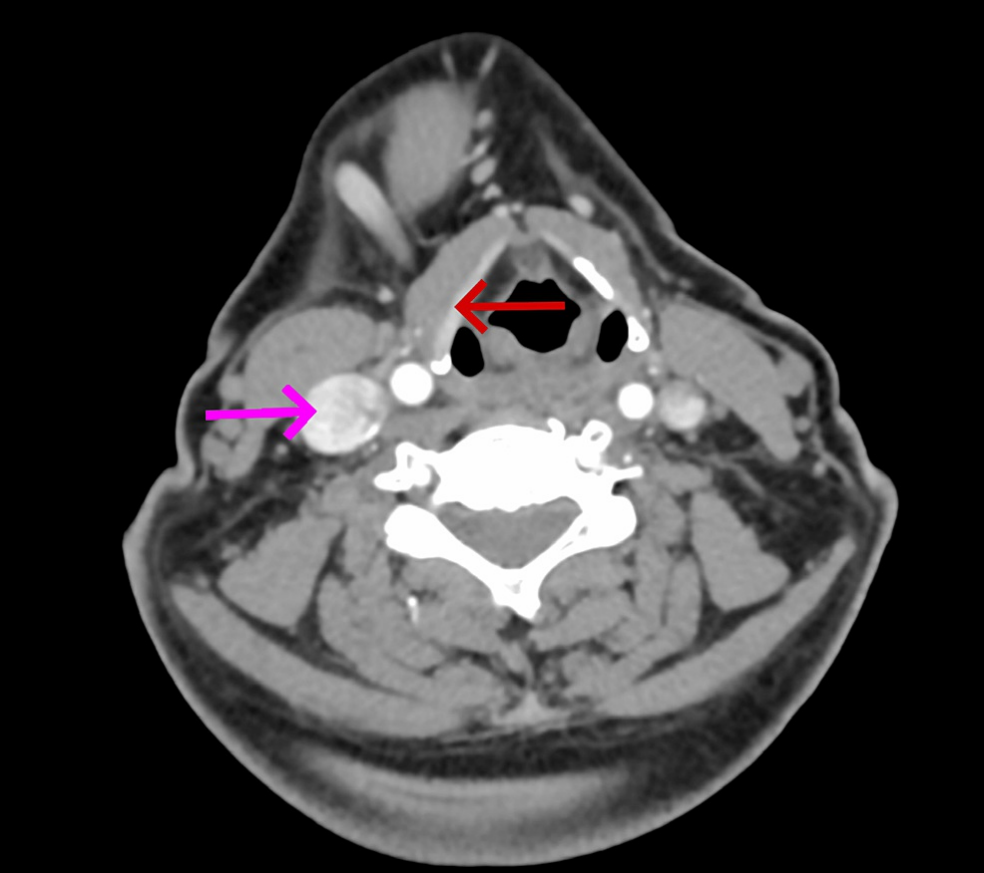
CECT of the neck CECT of the neck shows a well-defined lobulated mass with irregular margins and cystic areas measuring 3.5*3.5*5cm in the right submandibular region extending up to the midline (shown in pink arrow). Also non-visualized thyroid parenchyma suggestive of congenital hypoplastic thyroid (shown in red arrow). CECT - contrast-enhanced computed tomography

On local examination, there was a single firm mobile swelling in the right submandibular region measuring 5*4 cm and was non-tender. All relevant investigations were done and had normal thyroid function studies. Ultrasonography of the right submandibular region showed an enlarged submandibular gland with a hypoechoic lesion and vascularity on Doppler, suggestive of submandibular sialadenitis (Figure 1). FNAC of the swelling was done, and it showed numerous small basaloid cells in singles and multilayered. These cells have round to regular/oval nuclei with bland, granular chromatin. The background shows a few naked nuclei with fibrous scanty chondromyxoid stroma and hyaline material. These features are suggestive of a basaloid neoplasm like basal cell adenoma or pleomorphic adenoma. Contrast-enhanced computed tomography (CECT) of the neck showed a large lobulated mass with irregular margins and cystic areas in the right submandibular region extending up to the midline; post-contrast showed heterogenous enhancement with necrosis and coarse calcific foci within the lesion, suggestive of malignant neoplasm such as adenoid cystic carcinoma with origin from the deep lobe of the submandibular gland (Figure 2). Another small enhancing lesion with coarse calcific foci was seen at the left pharyngeal mucosal space, suggestive of a metastatic lingual lymph node. Also, the thyroid gland was not visualized, and a small oval isodense lesion with homogenous post-contrast enhancement was seen in the left lateral aspect of trachea, in the expected location of thyroid, suggestive of remnant thyroid/congenital hypoplastic thyroid. Thus the patient was advised surgical excision of the swelling.

**Figure 3:**
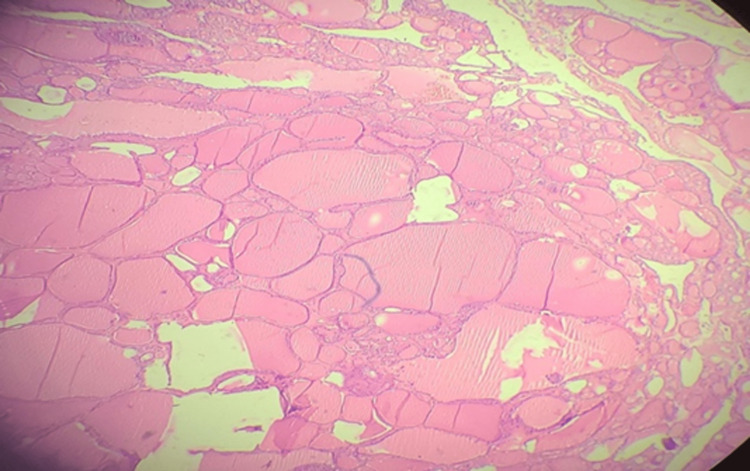
Biopsy of the operative specimen The biopsy shows well-encapsulated thyroid tissue composed of follicles of varying size, lined by low cuboidal to flat epithelium, and the follicles filled with proteinaceous material (H&E 40x).

After proper optimization and pulmonology, cardiology, and anesthesia clearance, the patient was taken up for right submandibular sialadenectomy. A right submandibular transverse incision, 2cm below the mandible was made. The right submandibular gland was found to be enlarged and infiltrating the surrounding structures. Sialadenectomy was done, hemostasis was attained, no. 10 drain was introduced, and the skin was closed in layers. The biopsy of the operative sample showed nodular colloid goiter with no metastatic foci in any sample and chronic sialadenitis which is a rare presentation (Figure 3).

## Discussion

Ectopic thyroid tissue is defined as the one which is located outside its normal anatomical position, anterior to the laryngeal cartilages, resulting from developmental defects during the early phases of thyroid gland embryogenesis. The most common location for ectopic thyroid tissue is lingual, but it can be present in other rare sites such as the mediastinum, esophagus, visceral organs, and intra-tracheal and submandibular regions, like our patient presented with [7]. Many cases of ectopic thyroid tissue present with an incomplete or poorly developed capsule. These tissue masses, even if benign, have been seen to invade the other adjacent structures, which was seen in our case as well, but it signifies an incompletely formed capsule.

Our patient presented with a slowly enlarging asymptomatic mass of the right submandibular region, which is similar to a few other cases that have been reported [8,9]. It’s also typical in that the lateral aberrant thyroid tissue in our patient was present in the submandibular region (70% of cases). The absence of a normal thyroid gland in the orthotopic position in our patient is also similar to most other cases (59%). Most cases of lateral aberrant thyroid are diagnosed definitively post-operatively (76%) after the surgical resection. In such cases, patients lose their only functional thyroid tissue, and it puts them at risk of hypothyroidism, which our patient also unfortunately suffers from. Post-operative thyroid function test result was consistent with compensated hypothyroidism; hence, she was put on thyroid replacement therapy.

The diagnosis of ETT is confirmed by the biopsy of the operative specimen. The different imaging modalities and clinical tests often fail to differentiate between a benign and a malignant ETT [10]. The usually performed radiological investigations such as USG and CT scan help in describing the extent, location, and consistency of the mass, but a biopsy is mandatory for confirming the diagnosis. A thyroid function test should be done both pre-operatively and post-operatively and is an essential test that should be mandatory in all cases of ETT [11]. In our case on FNAC, features of basaloid neoplasm like pleomorphic adenoma were seen as the lesion was evaluated as a submandibular mass and not thought of as a thyroid lesion at all. No thyroid tissue was obtained in FNAC; hence, The Bethesda System for Reporting Thyroid Cytopathology (TBSRTC), which consists of the following categories: I - non-diagnostic, II - benign, III - atypia of undetermined significance (AUS)/ follicular lesion of undetermined significance (FLUS), IV - follicular neoplasm/suspicious for follicular neoplasm (SFN), V - suspicious for malignancy, and VI - malignant; is not applicable here. Clinically it was not thought of as a case of ETT, instead was considered as a lesion of the submandibular gland. CECT was also pointing toward a malignant lesion of the submandibular gland, such as adenoid cystic carcinoma. FNAC was misleading as no thyroid tissue was obtained and showed features of a basaloid neoplasm. Finally, the definitive diagnosis of ETT was made from the histopathological examination of the operative specimen, which showed chronic sialadenitis with nodular colloid goiter with no metastatic foci.

As there is a risk of iatrogenic hypothyroidism in these patients, the decision of surgical resection of ETT should be based on the following - the age of the patient along with the symptoms, the progression of the swelling, results of the various diagnostic tests, and FNAC. In cases where a pre-operative accurate diagnosis of ETT is made, the ETT should be preserved, and the patient should be put on hormonal suppressive therapy. In our case, the patient was pre-operatively euthyroid and hence the diagnosis of ETT was not at all suspected. Surgery is preferred for cases that fail to respond to medical management or show features of malignancy [12].

## Conclusions

Ectopic thyroid is a rare clinical entity, but it should be kept as a differential diagnosis in cases of lateral aberrant thyroid. Pre-operative investigations are not definitive and often fail to detect the presence of ETT. Post-operative biopsy of the operative specimen is always definitive, but often it leads to the loss of the only functional thyroid tissue; hence, patients suffer from iatrogenic hypothyroidism. We presented a case of ETT of the right submandibular region simulating chronic sialadenitis, which was diagnosed post-operatively and treated with hormonal replacement therapy.
